# Multiphasic Multidetector Computed Tomography Study of a Rare Tracheal Tumor: Granular Cell Tumor

**DOI:** 10.1155/2014/807430

**Published:** 2014-12-08

**Authors:** Tommaso Guarnieri, Luciano Cardinale, Gianluca Macchia, Giancarlo Cortese, Andrea Veltri

**Affiliations:** ^1^Department of Radiology, San Luigi Gonzaga University Hospital, University of Torino, Regione Gonzole 10, 10043 Orbassano, Italy; ^2^Department of Radiology, Maria Vittoria Hospital, Via Cibrario 72, 10144 Torino, Italy

## Abstract

Our aim is to present the case report of a woman affected by tracheal granular cell tumor analysed by multiphasic contrast-enhanced multidetector CT. The tumor presents as polypoid lesion (diameter 13 mm), with smooth and well-defined margins, elevated contrast enhancement in arterial phase, and a modest release of contrast in venous phase. This pattern is quite different from the other tracheal tumours. We have performed a comprehensive review of literature to assess all cases of granular cell tumors of the trachea; only 40 cases are reported. Of these, no one focused on the contrast enhancement aspect, so our work is the first showing a specific pattern in multidetector computed tomography (MDCT) of the tracheal granular cell tumour and may help in differential diagnosis.

## 1. Case Report

A 64-year-old female, complaining of progressive inspiratory dyspnoea, was brought to the emergency department of our hospital. In anamnesis only a history of heavy smoker (20 pack-year) was reported.

Chest X-ray showed signs of congestion with redistribution of pulmonary blood flow (not shown).

The patient was treated with O2 therapy, diuretics, and antihypertensive drugs obtaining only slight improvement so she was hospitalized.

**Figure 1:**
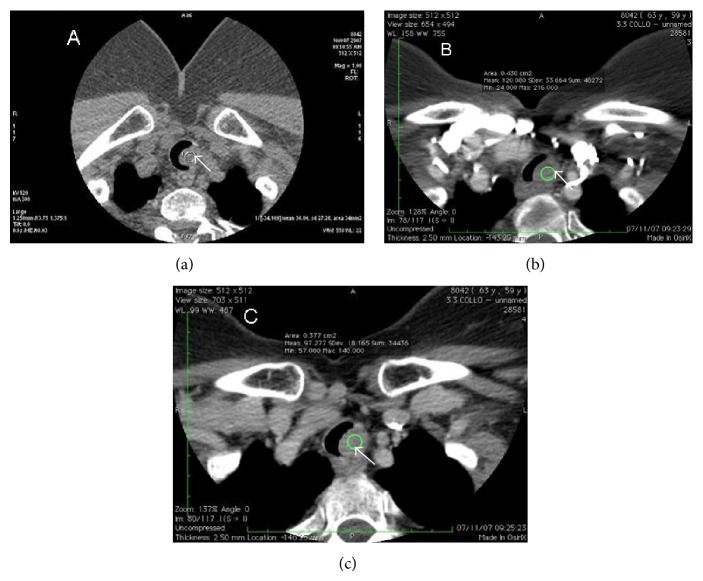
64-year-old female with granular cell tumor of trachea. Axial multiphase contrast-enhanced CT (16-slice Multidetector CT, GE Medical Systems) demonstrating polypoid lesion (diameter 13 mm) (white arrows), which presents smooth and well-defined margins and occupies the left side of the tracheal lumen. This mass does not present infiltrative signs suggestive of malignancy. The lesion shows elevated contrast enhancement in arterial phase and a modest release of contrast in venous phase; this behaviour is the tomographic expression of the rich vascularity of this kind of tumor. (a) Basal: 36 HU. (b) Arterial phase: 120 HU. (c) Venous phase: 100 HU.

**Figure 2:**
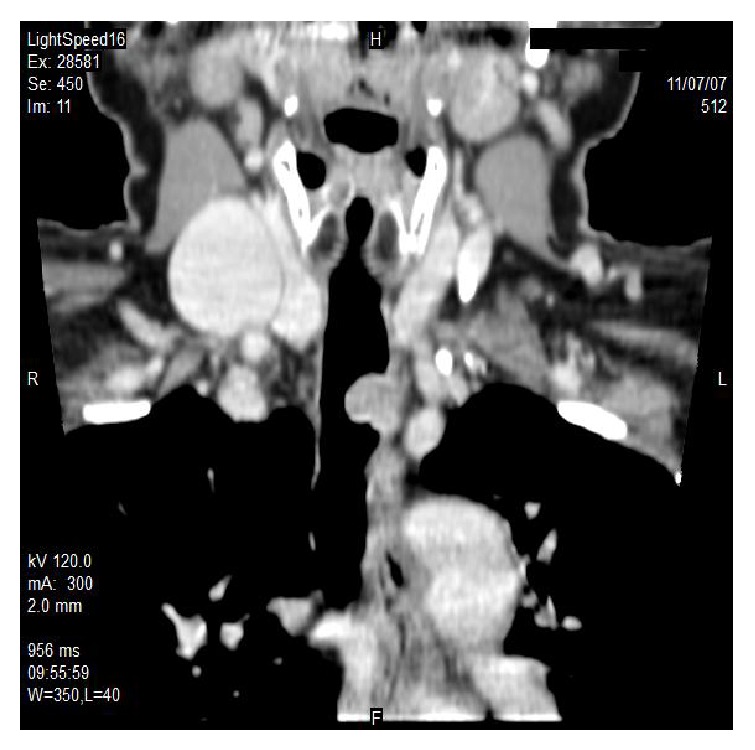
64-year-old female with granular cell tumor of trachea. Coronal contrast-enhanced CT scan (16-slice Multidetector CT, GE Medical Systems, venous phase) obtained at the level of the aortic arch shows an eccentrical mass (white arrow) growing in the left side of trachea, occupying half of the tracheal lumen.

**Figure 3:**
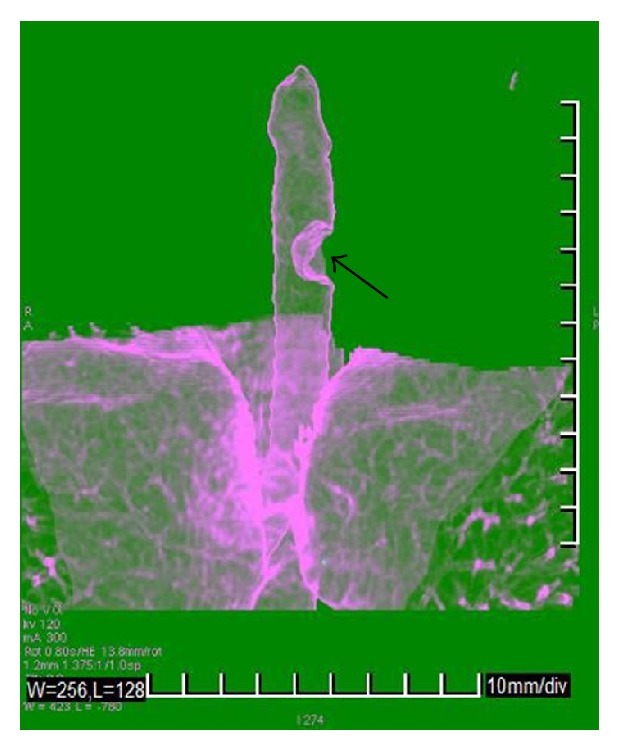
64-year-old female with granular cell tumor of trachea. Shaded surface display (SSD) image of trachea that shows the impact of mass (black arrow) on trachea lumen (16-slice Multidetector CT, GE Medical Systems).

**Figure 4:**
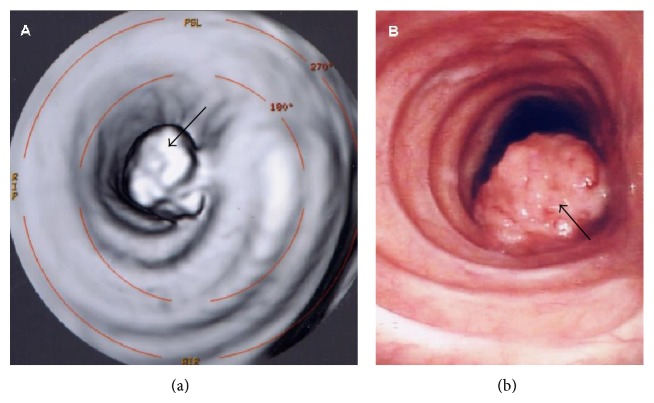
64-year-old female with granular cell tumor of trachea. (a) Virtual bronchoscopy image (16-slice Multidetector CT, GE Medical Systems) and (b) real bronchoscopy image that shows intratracheal sessile lesion (black arrows), characterized by round shape and polylobulated border, originating from left wall of middle portion of trachea and protruding into trachea lumen occupying half of its diameter.

We performed a preliminary precontrast CT (16-slice Multidetector CT, GE Medical Systems), which identified an intraluminal tracheal lesion of 13 mm, characterised by low density (36 HU), which occupied half diameter of tracheal lumen. The mass had smooth and well-defined border without signs of infiltration. Multiphase study showed elevated contrast enhancement in arterial phase and a modest release of contrast in venous phase (arterial phase: 120 HU; venous phase: 100 HU); this behaviour reflects the rich vascularity of this kind of tumor (Figures 1, 2, 3, and 4).

To assess the histology of the lesion, a biopsy was made during bronchoscopy (Figure 4(b)). The biopsy specimen was consistent with benign granular cell tumor.

The patient received endobronchial therapy (electrosurgery) and the clinical symptoms resolved. The follow-up CT did not show recurrence of disease.

## 2. Discussion

Granular cell tumor (also known as “Abrikossoff's tumor,” “granular cell myoblastoma,” “granular cell nerve sheath tumor,” and “granular cell schwannoma”) is a neoplasm of neural origin.

The neoplasm can affect all parts of the body with head and neck accounting for 45% to 65% of the cases (of that 70% are located in the tongue and 10% in the larynx).

Granular cell tumors are also found in the internal organs, particularly in the upper aerodigestive tract [1].

The usual presentation is of slow growing tumor. Granular cell tumors are typically solitary and are rarely larger than three centimeters. This type of tumor can be both benign and malignant, although malignancy is rare and comprises only 2% of all granular cell tumors [2]. Granular cell tumor characteristics are summarized in Table 1.

Our revision of English literature has shown only 40 cases of granular cell tumor of trachea and in all the cases only clinical characteristics but not radiological images were investigated [1–8].

Our study showed a quite different pattern in comparison to the other benign tumours of trachea. The preliminary phase without contrast administration suggested the benign nature of the lesion (smooth margin, no sign of invasion) and the arterial and venous phase (elevated contrast enhancement in arterial phase and a modest release of contrast in venous phase) showed differences with respect to the other benign tumour of trachea that is useful for differential diagnosis (Figure 1).

Tumors in the tracheobronchial tree are rare, accounting for less than 0.4% of all body tumors [9].

However, the vast majority of tracheobronchial tumors in adults are malignant (squamous cell carcinoma (most common), adenocarcinoma, neuroendocrine tumors (large cell neuroendocrine tumors, small cell carcinoma), adenoid cystic carcinoma (second most common), sarcoma, malignant lymphoma, and metastases) although various benign tumors may also occur in the tracheobronchial tree.

Benign tumors of trachea are about 10% of total tracheal tumors [9].

Granular cell tumor, compared with the other benign tumors of the trachea, differs in the following aspects.

(i) Hamartoma is rarely localized in trachea (in the literature hamartoma has been reported only in about ten cases); its most frequent localisation is pulmonary.

At CT this kind of tumor is characterized by low density for high adipose content and in 25% of cases shows the “popcorn” calcifications [10].

(ii) Hemangioma is more common in children, localised in trachea, and often manifests with hemoptysis [11]. It is also characterized by a rich enhancement in the arterial phase, but with filling from the periphery toward the center of the lesion.

(iii) Tracheobronchial leiomyoma occurs most commonly in the fourth decade of life, although one-third of patients are younger than 20 years old. Approximately 45% of leiomyomas are endobronchial; the rest occurs in trachea and lung.

Leiomyomas usually are small and are found incidentally during bronchoscopy performed to evaluate other lung lesions.

The lesion appears as an airway intraluminal nodule with oval, lobulated, or round edge. The internal content of tumor is homogeneous with homogeneous enhancement.

This tumor has an attenuation of 25–46 HU on unenhanced CT and 46–85 HU on enhanced CT (less enhancement than Abrikossoff's tumor).

The rare big leiomyomas (over 40 mm) can be heterogeneous with stippled calcifications and on enhanced scans these tumors have heterogeneous enhancement (this big tumor has intraluminal and extraluminal components) [12].

(iv) Lipoma of tracheobronchial tree is very rare (0.01% of all bronchial tumors) and it is usually found endobronchially. In the majority of cases, it is located in the first three subdivisions of the tracheobronchial tree.

It has characteristic density which is very different from other tracheal tumors (from −50 to −100 HU) [13].

(v) Carcinoid of the trachea is an extremely rare entity with very few cases reported in the literature, 20 cases reported in Chinese literature and 15 cases reported in English literature [14]. Carcinoids have a rich vascularity (intense arterial phase) but cause earlier symptoms than granular cell tumor because they are more often localized in bronchial tree than in trachea, and for this reason carcinoids cause early obstruction and air trapping, mucoid stagnation, bronchiectasis, and pneumonia.

Moreover, carcinoids in 30% of cases have calcifications which are not present in the granular cell tumors.

Differential diagnoses are summarized in Table 2.

## 3. Conclusion

The CT aspect of benign tracheal tumors helps in differential diagnosis. The association of the epidemiology, the site of origin, the morphology, and in particular the characteristics of enhancement with multiphase approach can suggest the diagnosis of the nature of the tumor that must be confirmed with histological specimen.

## Figures and Tables

**Table 1:** Summary of granular cell tumor of trachea.

Etiology	Schwann cell origin
Incidence	Very rare, 40 cases reported in literature
Gender	Most granular cell tumors are found in females
Age predilection	Third and fourth decades of life
Risk factors	Unknown (hyperestrogenic state had been hypothesized)
Treatment	Chirurgy
Prognosis	Good with successful resection
Finding on imaging	Smooth and well-defined margins, elevated contrast enhancement in arterial phase, and a modest release of contrast in venous phase

**Table 2:** Differential diagnosis table for granular cell tumor of trachea.

Entity	CT findings
Hamartoma	“Popcorn” calcifications; low density for adipose content

Hemangioma	Rich enhancement in the arterial phase, filling from the periphery toward the center of the lesion

Tracheobronchial leiomyoma	Oval, lobulated, or round contour, homogeneous content with homogeneous enhancement. 25–46 HU on unenhanced CT and 46–85 HU on portal phase

Lipoma	HU from −50 to −100 (density of fat)

Carcinoid	Rich enhancement in the arterial phase (150 HU). Calcification (30% of cases)

## Abbreviations

MDTC: Multidetector computed tomography
CT: Computed tomography
HU: Hounsfield unit
GE: General Electric
mm: Millimetre
MIP: Minimum intensity projection
SSD: Shaded surface display.
